# Efficacy and community-effectiveness of insecticide treated nets for the control of visceral leishmaniasis: A systematic review

**DOI:** 10.1371/journal.pntd.0010196

**Published:** 2022-03-02

**Authors:** Carlos Alberto Montenegro-Quiñonez, Claudia Buhler, Olaf Horstick, Silvia Runge-Ranzinger, Kazi Mizanur Rahman

**Affiliations:** 1 Heidelberg Institute of Global Health, Research to Practice Group, Heidelberg University, Heidelberg, Germany; 2 Instituto de Investigaciones, Centro Universitario de Zacapa, Universidad de San Carlos de Guatemala, Zacapa, Guatemala; 3 The University of Sydney, University Centre for Rural Health, Lismore, New South Wales, Australia; Institute of Continuing Medical Education of Ioannina, GREECE

## Abstract

Visceral leishmaniasis (VL) has been targeted for elimination from Southeast Asia (SEA). The disease has been endemic in SEA, and in other parts of the world involving both humans and animals. One of the key strategies for combating VL is controlling for the vector sandfly. There are a few vector control strategies that are currently in practice. We sought to assess the efficacy and community effectiveness of insecticide treated nets (ITNs) in controlling the burden of sandfly and the occurrence of VL among humans. We conducted a systematic review following a study protocol and the Preferred Reporting Items for Systematic reviews and Meta-Analyses (PRISMA) criteria. 6331 initial hits were retrieved from Google Scholar, Lilacs, PubMed, Science Direct, WHOlis, WHOiris and PAHOiris. 25 met the full inclusion criteria. Findings show that the insecticide impregnated bednets and the commercially treated long lasting insecticidal nets (LLINs) are effective in controlling sandflies, with mortalities as high as 75% lasting over a year; although their role in controlling VL in the community was not extensively studied, since effectiveness was usually measured with sandflies densities. Findings also show that insecticide impregnated bednets are low cost and well accepted in the community, however, early erosion of insecticides from nets could occur. Some studies also showed that killing of sandflies may not translate into reduction of VL, therefore sandfly knock down and killing data needs to be interpreted with caution. Conclusions of this review are (1) combining insecticide impregnated bednets, as targeted interventions, with another vector control measure, particularly indoor residual spraying, and in conjunction with case detection, could be the way forward to controlling VL in resource limited settings. (2) Given the current low incidence of VL in SEA, it can be difficult to further research the community effectiveness of those control measures in reducing VL.

## Author summary

Visceral leishmaniasis (VL) is a vector borne disease endemic in Southeast Asia (SEA) and present in other regions of the world. In SEA the disease has been targeted for elimination. VL is transmitted by different species of sandflies and key strategies against VL rely on the control of the vectors. This systematic review assesses the efficacy and community effectiveness of insecticide treated nets (ITNs) in controlling sandflies and VL occurrence among humans. After a revision of 6331 initial hits, 25 studies were included in this systematic review. Main findings indicated that insecticide impregnated bednets and long-lasting insecticidal nets (LLINs) can effectively control sandflies, however, it was not possible to fully assess the role of ITNs in controlling VL transmission. Additional results showed that insecticide impregnated bednets are low cost and well accepted in the community, however early erosion of insecticides from the nets could occur. If insecticide impregnated bednets are combined with other vector control measures, such as indoor residual spraying, and together with case detection, it could be an important strategy to control VL in resource limited settings.

## Introduction

Visceral leishmaniasis (VL), also known as kala-azar, as a Neglected Tropical Disease of poverty, has been targeted for elimination from Southeast Asia (SEA) [1]. Efforts have also been made to control for the disease in other parts of the world, where VL can involve both humans and animals [2].

As per a 2012 estimate, there were 200,000 to 400,000 VL cases from 79 countries and 20,000 to 40,000 deaths annually in the previous five years [3]. Eighty percent of global VL cases were then reported from the South Asian eco-epidemiological hotspot comprising countries including Bangladesh, India and Nepal. More recent data show that globally 23,804 cases were reported to WHO in 2015, of which 9,249 (39%) were from the South Asian hotspot [4]. Annual cases of kala-azar in these South Asian countries declined from 77,000 cases in 1992 to fewer than 7000 in 2016 [5]. Sporadic cases are also reported from the other countries in the SEA region including Bhutan, Myanmar, Sri Lanka and Thailand.

VL is transmitted by different species of sandflies, mainly the vectors *Phlebotomus argentipes* and different species of the sandflies *Lutzomyia* [2,5]. Among the strategies being in place for prevention, control and elimination, effective vector control is one [6]. In different parts of the world, in different settings, varying vector control measures are in place [7]. Investigations have been conducted to understand the effectiveness of those measures using a wide range of study designs in various settings and on different vector species. A recently conducted meta-review by Montenegro et al. on vector control measures for both VL and cutaneous leishmaniasis (CL) have found significant research gaps [7]. Gaps were specifically identified in the availability of robust and comparable study designs, and comparability of settings and interventions. Human outcome parameters of vector control measures were missing and the majority of studies were embedded in programme assessments, meaning that the single interventions are not sufficiently assessed individually and the combination of interventions not assessed systematically.

In this systematic review, we focused on insecticide treated nets (ITNs) and its efficacy and community-effectiveness for the control of VL and its vectors globally. We present our findings with recommendations for policy, identify critical gaps in evidence base and for further research studies.

## Methods

### Search strategy, databases and search terms

In response to the above-mentioned meta-review of vector control interventions for the control of CL/VL [7] and the subsequent gap-analysis, this systematic review has been undertaken along with other systematic reviews for different vector control interventions. The present study focuses on ITNs, including bednets, to control VL. Key difference to previously published work is the inclusion of studies with randomisation, but also those without randomisation, considering that a lot of studies analysing vector control methods have been undertaken without randomisation processes.

As a first step a study protocol was established. The systematic review follows the Preferred Reporting Items for Systematic reviews and Meta-Analyses (PRISMA) criteria [8].

All searches have been carried out until 17 of October 2020, using the following databases: Google Scholar (considering the database is sorted by relevance, we screened the first 200 hits only, after establishing that for most of our searches no relevant hits were found after screening the first 100 hits), Latin American and Caribbean Health Sciences Literature (LILACS), PAHOiris, PubMed, ScienceDirect, WHO Library Database (WHOlis) and WHOiris.

The searches were performed without restriction to language, publication year or region of publication. Searches were performed in English with a focus on primary research articles with no restriction to a specific study design.

1. Disease: “Visceral Leishmaniasis” (MeSH terms and “major topic” function were used where supported by the database)
2. Intervention: “ITN” or “Insecticide treated nets” and its potential variations (MeSH terms were used, where supported by the database)
3. Vectors for transmission of visceral leishmaniasis were used as free-text terms.
4. Inclusion criteria were all studies focusing on 1) Visceral leishmaniasis 2) Insecticide treated nets or synonyms, and 3) all primary research methods were included.

Exclusion criteria were conference or opinion articles, editorials or any articles without clear primary research methodology. Furthermore, the reference lists of all included articles were manually searched for additional articles. As for grey literature, guidelines, not older than 5 years, for VL were screened, including reference lists.

All searches performed have been documented, including the selection process. Two data extractors (CAMQ and CB) independently screened titles and abstracts and applied inclusion and exclusion criteria. In case of disagreement a third researcher (SRR) was involved to reach consensus.

The included studies have been categorised for further analysis into the following categories:

1. Research articles focused on ITN interventions only
2. Research articles focused on comparing ITN with at least one other intervention

Finally, each of the above-described categories are divided 3) into the subcategories a) interventional [cluster randomised controlled trial (cRCT), randomised controlled trial (RCT), non-randomised controlled trial and community intervention trials] or b) observational study designs. We also aimed to analyse according to geographical regions, due to the different vectors involved in the context of VL.

### Quality assessment

Quality assessment has been performed using the CONSORT checklist [9] for a) RCTs and cRCTs, for b) other studies, CONSORT has been used, excluding the criteria for randomisation. The CONSORT checklist for RCTs is composed of 25 items, a high-quality study would have most of the items fulfilled in the study, for example, a study fulfilling 25 out of the 25 items of the checklist would have a quality equal to 100%. For other studies, the elimination of the randomisation criteria from the CONSORT checklist resulted in a list of 18 items. In this case, a high-quality study, not being RCT or cRCT, would have most of the 18 items of the checklist fulfilled. Studies have not been excluded following the quality assessment, but analysis and reporting reflected the quality assessment.

### Data extraction and analysis

Data have been extracted in a predefined data extraction matrix and analysed by author, year of publication, country/geographical region, study type, methods used, sample size, follow up period, study arms and interventions included, different vectors and entomological outcome indicators, human outcome indicators, reported conclusions, limitations and quality assessment scores. For p-values, 5% level of significance was used for all the studies. Evidence tables were developed for presentation of data and analysis.

## Results

### Descriptive results

#### Results of searches

**Fig 1:**
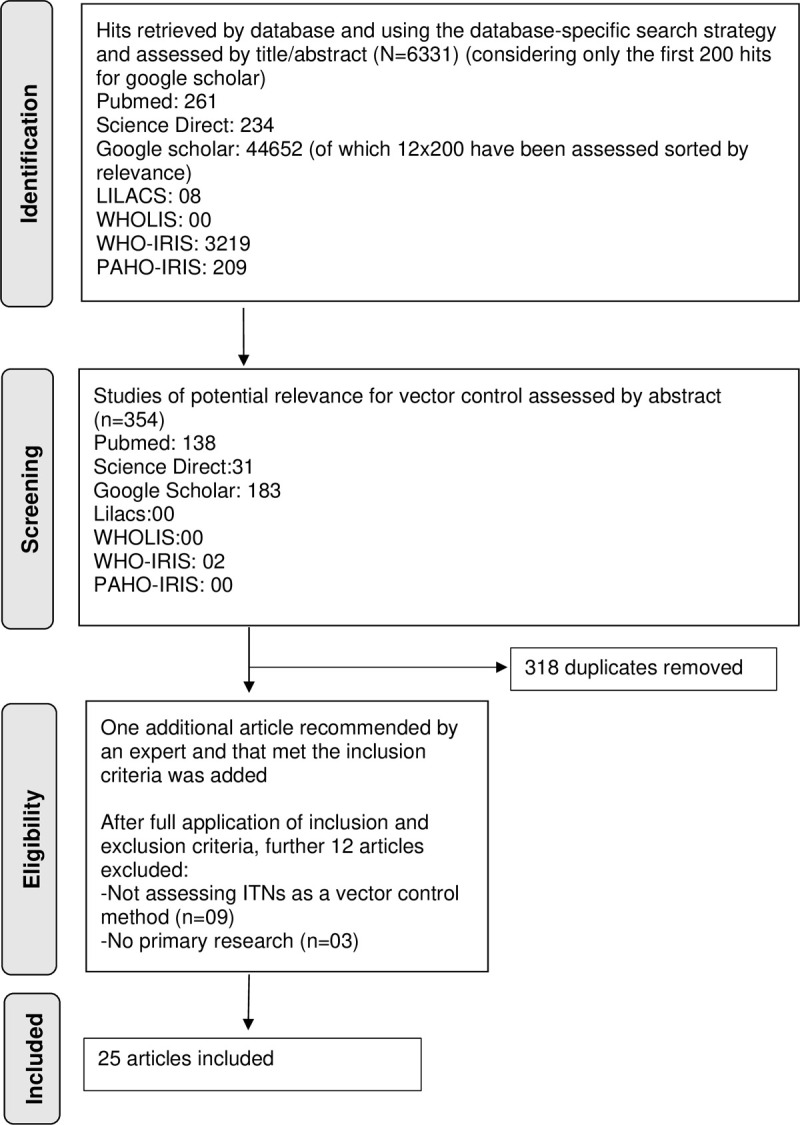
PRISMA flowchart for searches and inclusion of articles for Systematic Review on the use of insecticide treated nets for visceral leishmaniasis control.

6331 initial hits were retrieved on the seven included databases. After assessing by title and abstract, 354 articles were further screened. Removing 318 duplicates 37 articles were fully assessed, and 25 were included after full application of inclusion and exclusion criteria. (see Fig 1). Of the 25 finally included studies, 22 were retrieved on PubMed, 2 on Google Scholar, and one additional recommendation by an expert that met the inclusion criteria was added (Table 1).

**Table 1:** VL ITN evidence table.

Author, Year, Country (District/subdistrict), study design	Sample size/Follow up period	ITN interventions			Vector/parasite species	Results: Human outcome indicators			Results: Entomological outcome indicators			Limitations described	Quality assessment scores by the authors (CONSORT checklist)
		Bed nets impregnation with KO Tab 1-2-3	Long-lasting insecticidal nets	Combinations		Perception, acceptability, access, utilization, maintenance and satisfaction regarding ITNs	Sandfly exposure	Incidence of L.donovani infection /seroconversion/VL incidence	Sandfly density	Sandfly mortality	Insecticide residue on ITNs, sandfly landing and knock down of sandflies		
**Cluster Randomised Controlled Trials (cRCT)**													
Huda 2019 Bangladesh (Mymensingh district, Fulbaria and Trishal subdistricts) cRCT [19]	The total number of HHs for the FC+ITN intervention was 8143 (36,869 pop.) The total activity period was of 18 months; 4 months of pre-intervention, 2 months of intervention and 12 months of follow-up activities Sandfly density was evaluated 2–3 days before starting the intervention and follow-up surveys at 1, 3, 6, 9, and 12 months	✓			*Phlebotomus argentipes*				At baseline, the average female *P*. *argentipes* density per household was about 0.22 (SD, 0.48) in the FC + ITN intervention. The mean reduction of female *P*. *argentipes* density in the ITN arm ranged from −6.10 (−3.04, −9.19 CI) to 0.81 (0.01, 1.60 CI). The adjusted intervention effect of ITN was statistically significant on reduction of the incidence rate of female *P*. *argentipes* sandfly count up to 3 months post-intervention but not beyond follow-ups.	Efficacy based on WHO cone bioassay: The corrected mortality of *P*. *argentipes* sandfly was 51.7%, on insecticide-treated bed-nets at the 12 month follow up.		The study had fewer index cases than planned, which meant that only two of the three arms (NKTA and FC + ITN) had the minimum calculated 18 index case–based interventions. They could not have an epidemiological end point because of the currently low incidence of VL in Bangladesh; therefore, they could not measure the effects of reduced vector densities on parasite transmission and new infections and cases.	17/25
Mondal 2016 Bangladesh, Nepal and India Multi-country cRCT [20]	21 villages were selected (13, 4 and 4 from Bangladesh,India and Nepal respectively). A total of 3667 HHs were part of the study Sand fly density was measure 2 weeks before the intervention and the follow up period was at 1, 3, 9 & 12 months.	✓			*Phlebotomus argentipes/ Leishmania donovani*	94% DWL and ITN. Transient itching was reported by 4.8% of HHs with ITN. Unpleasant smell was reported in 3.2% of ITN houses.			For the effect of sand fly density, at baseline, sand fly density was similar in intervention and control HHs. In the ITN clusters the sand fly density was also reduced, however the effect only lasted through 9 months.	For the effect on sand fly mortality. The Abbott’s corrected sand fly mortality rate [mean and 95%CI] at 1, 3, 9 and 12 months after interventions was respectively 75% (71%-79%), 67% (64%-74%), 63% (57%-68%) and 49% (43%-55%) for ITN.		The study showed a reduce duration of efficacy (9 months) compared to the duration (18 months) reported in previous studies in Bangladesh. Which could be explained by operational errors during bed net impregnation and the breakdown of insecticide in nets over time. Commercial LLINs are advantageous in this regard as they are less susceptible to operational errors and rapid insecticide loss. The major limitation of the study is the inability to use an epidemiological endpoint due to low of VL. To attend this, in order to expect such an effect demonstration of reduced sand fly density and increase mortality is necessary, which were the aim of the present study.	20/25
Picado 2010 India and Nepal Paired cRCT [21]	26 high incidence clusters (a complete hamlet or a ward) (16 in India, 10 in Nepal). 13 clusters were allocated to intervention (India 8; Nepal 5) with 9829 participants 13 clusters were allocated to control (India 8; Nepal 5) with 9981 participants Intervention: Between November and December 2006 Follow up period: November- December 2007 and 2008. And from November 2006 to May 2009 for quarterly house to house surveys.		✓		*Phlebotomus argentipes /Leishmania donovani*			The risk of seroconversion during the two-year follow-up was significantly different between countries: 7.2% in India (529/7368) and 3.1% (163/5323) in Nepal. The overall risk of seroconversion in the intervention (5.4%) and control (5.5%) groups was similar. At cluster level, the risk of infection was reduced by 10% in the intervention clusters compared with control clusters, but this effect was not significant. Longlasting insecticidal nets (LN/LLIN) seemed to have an opposite effect on seroconversion in India and Nepal but the interaction was not significant. The overall risk of visceral leishmaniasis (VL) during the two-year follow-up was 0.38% and 0.40% in the intervention group and control group, respectively. The cluster level analysis showed that longlasting insecticidal nets reduced the risk of visceral leishmaniasis by 1%, but the effect was not significant.	Indoor density of *P*. *argentipes* was reduced by 25% in the study clusters that used treated nets compared with control clusters.			The incidence of Leishmania infection in study clusters could have been lowered by the active detection and treatment of cases of visceral leishmaniasis implemented during the trial but did not fall below the assumption of 2% annual incidence made when calculated the sample size. There was a slightly higher proportion of lost to follow-up in control (21%) than in intervention (19%) clusters, however the research team do not expect this to affect the estimations as the characteristics of these people were similar.	21/25
Picado 2011 India (Bihar) and southeastern Nepal Community randomised trial [22]	November–December 2006 4300 PermaNet 2.0 were distributed in 13 VL endemic villages (8 in Bihar, India and 5 in southeastern Nepal) (November–December 2006). Information on washing behaviour and LN use was gathered from 578 and 642 households at 6 and 22 months post-distribution of LNs respectively. The physical condition of the LN was assessed after 6 and 22 months of household use.		✓ PermaNets (baseline deltamethrin concentration levels above 55 mg		*Phlebotomus argentipes* (Bioassays were done on *Anopheles gambiae)*	After 22 months of use, 36.6% of nets in India and 22.2% in Nepal were still intact.					After 12 months of use the deltamethrin concentration per LN was reduced to an average of 30.1 and 36.7 mg/m2 in India and Nepal, respectively. After 24 months there was further loss which was greater in India (11.6 mg deltamethrin/m2 remaining) than in Nepal (27.9 mg/m2 remaining). The mean Knock down effect (KD) per swatch ranged from 88.5–99.0% after 12 months and 87.6–99.0% after 24 months. The mean mortality per swatch ranged from 91.0–100.0% after 12 months and 87.6–100.0% after 24 months. All LNs, from both countries, scored higher than the failure threshold (KD≥95% or mortality≥80%) after 24 months of household use.	The data on the number of washes used to evaluate the HPLC results should be interpreted with a degree of caution, as the value for washing frequency was that reported by the head of household rather than by the person actually using the LN taken for testing. Since the LNs tested with bioassay were not randomly selected, these results probably underestimate the mean insecticidal effectiveness of LNs at the end of the trial.	16/25
Gidwani 2011 India and Nepal Paired cRCT [23]	The blood samples included in this study are a subset of the samples collected in the study from Picado, et. Al. 2010 [21] For this study, 305 individuals were selected from both cluster of the study (150 intervention and 155 control). Follow up period: 12 and 24 months post-intervention.		✓		*Phlebotomus argentipes* and the sympatric (non-vector) *Phlebotomus papatasi* */Leishmania donovani*		For *P*. *argentipes* the geometric mean of ELISA OD was on average 12% reduced at 12 months and 9% at 24 months in the intervention group compared to control group. Similar results were obtained for *P*. *papatasi*: 11% and 9% reduction in LN group at 12 and 24 months respectively. In addition, the percentages of positive samples for *P*. *argentipes* ELISA were reduced from 63.2% to 43.5% and from 47.1% to 43.6% in the intervention and control groups respectively over 24 months. For *P*. *papatasi*, the percentage of positive ELISA samples was not altered after 24 months in the control clusters (17%) but was reduced from 32.6% to 21.8% in the clusters using LN.					Regarding limitations: The baseline sand fly saliva antibody values were different between intervention and control groups; people in intervention clusters seemed to have a higher sand fly exposure before the LNs were distributed. This contrasts with the baseline data from the trial which showed that intervention and control clusters had similar indoor *P*. *argentipes* density and similar population characteristics. This difference may be due to random error as the number of samples per cluster was small (6 to 17 subjects/cluster) and there were some differences between groups at baseline.	17/25
Das 2010 Nepal (Sunsari and Morang) cRCT [24]	Nepal November 2006 to April 2007 The study area included two districts in eastern part of Nepal namely Sunsari and Morang, with 24 clusters (1335 HHs and 6955 habitants). The study was carried out from November 2006 to April 2007. Baseline and follow-up surveys were conducted on 2weeks before and 2 weeks, 4 weeks and 5 months after the interventions.		✓PermaNet		*Phlebotomus argentipes*, *P*. *papatasi and Sergentomyia spp*				For LLIN, vector density dropped from 7.9 to 0.9 per house per night by LT collections and from 1.8 to 0.5 per house per morning aspirator collections in three follow-up surveys after intervention.			During the study it was observed that the decline in sand fly density in all arms during first follow-up survey might be due to adverse environmental conditions; low temperature and humidity, etc. During the subsequent follow-up surveys, increasing tendency in sand fly density might be the influence of favourable environmental condition and successive loss of effectiveness of the intervention methods.	11/25
Joshi 2009 Bangladesh, India and Nepal cRCT [25]	24 clusters per site (consisting of 120 HH per site, Bangladesh, India, Nepal 1 and Nepal 2) For the follow up period: Sand fly collections were taken before the application of the interventions and follow-up sand fly collection were taken after 5-months for India and Nepal and 6-months for Bangladesh.		✓PermaNet nets with small mesh (156 holes/in2), polyester, resin coating containing deltamethrin (55 mg/m2)		*Phlebotomus argentipes* and the non or possible vectors *P*. *papatasi and Sergentomyia spp*.*/ Leishmania donovani*				The estimated intervention effect in terms of reduction in sand fly counts in the simple model showed a 72.4% reduction for IRS, a 42.0% reduction for EVM and a 43.7% reduction for LLIN. The estimated intervention effects for site specific analysis show a pattern were LLINs had a significant negative effect on sand fly densities in India and Bangladesh, but not in the two Nepal sites.			The site-specific data regarding LLINs and EVM have to be interpreted with caution because the limited number of clusters per arm provided uncertain estimates (wide confidence Intervals). For the results regarding the protective efficacy of LLINs, it has to be taken into consideration that CDC light trap captures monitor sand flies outside the bed nets, so that the actual protection for people sleeping under a LLIN from infective bites is most likely to be higher. An additional limitation is that this study was conducted under fairly controlled conditions which are not easily applicable in a national vector control programme. However, it provides an indication of the way in which to undertake treatment programmes.	16/25
Picado 2010 India and Nepal cRCT [26]	12 clusters (6 in Muzaffarpur district, India and 6 in Sunsari district, Nepal). This clusters are a subset of the 26 clusters of the study from Picado, et. Al. 2010 [21] For monitoring: Pre-intervention collections for 4 months in India and 3 in Nepal were done. And in both countries there were 12 monthly postintervention collections for follow up (September 2006 to November and December 2007)		✓		*Phlebotomus argentipes*, *P*. *papatasi and Sergentomyia spp*.				The principal finding from the analyses at a household level was that the cluster-wide distribution of LNs significantly reduced the 12-month post-intervention of total *P*. *argentipes*/house by 24.9% in intervention compared to control clusters. The effect was not significantly different in the two countries. The analyses carried out on *P*. *argentipes* females also found a significant reduction in density of 11.6%.			The low number of female *P*. *argentipes* captured by aspiration in households post-intervention did not allowed any formal statistical analysis. The overall blood feeding rates postintervention, being -52% in intervention and 31% in control groups, should be interpreted with caution as aspiration captures were clustered in only a few households. In addition, results based on aspiration captures in cattle sheds should be interpreted with caution as the number of *P*. *argentipes* collected was highly variable between and within clusters	19/25
Chowdhury 2011 Bangladesh (Fulbaria) cRCT [27]	The intervention trial was conducted in Fulbaria subdistrict, Mymensingh District. Four villages were randomly selected from the 20 villages, each village was divided into six geographic clusters (24 clusters). 120 HH were selected for vector collection. Pre-intervention vector collections were carried out in October 2006. For follow up: Post intervention collections were conducted in December 2006, January, March, April, and October 2007		✓ PermaNet 20		*Phlebotomus argentipes*, other *Phlebotomus spp*., *Sergentomyia spp*. */Leishmania donovani*				The proportion of gravid female *P*. *argentipes* ranged from 28% to 40% in the baseline survey. Post intervention, the proportion of gravid remained below 20% in the ITN arm.		In the baseline insecticide susceptibility testing, the sand fly knockdown rate at 1 hour was 88%, and mortality at 24 hours was 100%.	The researchers suggest a need to assess the impact on both entomological and disease factors, since the study focus only on entomological outcomes.	16/25
Chowdhury 2017 Bangladesh (Fulbaria) cRCT [28]	3079 houses from 11 villages divided into 10 sections with 6 clusters per section from the Fulbaria upazila (sub-district) of Mymensingh district (13406 inhabitants) There was a baseline measurement of vector density and follow up in 2, 4, 5, 7, 11, 14, 15, 18 and 22 months after intervention.	✓	✓	IRS+LLIN, IRS+KOTAB, LLIN+OUT, KOTAB+OUT	*Phlebotomus argentipes*				The adjusted model for LLIN showed that the vector density reduction was 9% to 78% and the rate ratio was between 0.91 and 0.32 for two years. In the KOTAB arm, a significant sand fly density reduction was observed only at the third, sixth, seventh and eighth follow-up measurements. For the other combined intervention arms: The combination of IRS with LLIN (IRS+LLIN) or with KOTAB (IRS+KOTAB) and with outdoor spray (IRS+OUT) showed statistically significant *P*. *argentipes* reductions throughout the study period except at the second follow-up for IRS+KOTAB. The adjusted model showed that the effects of IRS+LLIN and IRS+KOTAB were 23.0% density reduction to 85.0% and 16.0% to 86.0% respectively. The combination of LLIN with outdoor spray (LLIN+OUT) was found to be effective throughout the study period at a highly significance level. The reduction of *P*. *argentipes* sand fly density in this case was 26.0% to 86.0%. The combination of outdoor spraying with KOTAB (OUT+KOTAB) had no statistically significant effect on density reduction of *P*. *argentipes* sand fly counts in most of the follow-up points.	For the Sand fly mortality bioassays: The mortality for LLIN was 82.59% at 20 months of use. The mortality on K-O TAB 1-2-3 impregnated nets dropped from 88.37% at 3 months to 69.12% at 20 months after use.		The study did not evaluate the relationship between vector density (males plus females) and leishmaniasis incidence, which the researchers indicate that it should be investigated.	18/25
**Randomised Controlled Trial (RCT)**													
Banjara2019 Nepal (Saptari) RCT [29]	June to August 2016 Intervention –264 HHs Control—92 HHs Sandfly collection and density measurement: 1, 3, 9, and 12 months after intervention	✓			*Phlebotomus argentipes/ Leishmania donovani*	72% perceived a reduction of sandflies after bed net impregnation with insecticide			For sandfly density reduction: IRS and insecticide-impregnated bednets were effective for up to 1 month after application, respectively, but their efficacy waned thereafter. The bioassays performed on the treated surfaces showed that the mortality of *Phlebotomus argentipes* sandflies was about 50% and 26%, respectively, for insecticide-treated bednets; and 99% and 23%, respectively, for IRS.	The bioassays performed on the treated surfaces showed that the mortality of *Phlebotomus argentipes* sandflies was about 50% and 26%, respectively, for insecticide-treated bednets; and 99% and 23%, respectively, for IRS.		Within the study it was evident that the spatial effect of the intervention seems to be crucial to ensure the efficacy of bed net intervention. Since the bednet impregnation intervention was carried out only in some target houses, and thus, the community effect was lost.	15/25
Alexander 1995 Colombia (Cali) [Not from the South Asian regional initiative] Radomized matched design [13]	Three houses, assigned randomly to each treatment Six months–first trial Six months–second trial			✓ (Bednets and curtains impregnated with deltamethrin)	*Lutzomyia columbiana*, *Lu*. *Lichyi*, *Lu*.*youngi*		The proportion of *Lu*. *youngi* females biting was significantly lower in rooms protected by curtains (0.48 v 0.65, p<0.05) but not for *Lu*.*lichyi or Lu*. *columbiana*, *although they appeared to less predisposed to bite after passing through impregnated curtains*. Only nine *Lu*. *youngi*, one *Lu*. *Lichyi* and one *Lu*. *columbiana* were collected biting inside the impregnated bednets. The overall rate of 0.14 sandflies/man-hour was considerably less than that of 4.36/man-hour recorded for unprotected rooms.		The mean number of sandflies collected per man-hour in rooms fitted with impregnated curtains was not significantly different from the number caught in rooms without curtains.	*Lu*.*youngi* showed significant knockdown within 1 h (13.2%) and mortality after 24 h (43.4%) of exposure to impregnated curtains. *Lu*. *Youngi* collected resting on impregnated bednets showed a mean knockdown rate of 7.1% after 1h and mortality of 39.7% after 24h, significantly higher (p < .05) than the rates for those caught resting on untreated walls of houses without impregnated bednets or curtains.		The mesh size (64 per cm^2^) of the nylon netting used in the study was wide enough to permit sandflies to pass through. The buildings were not completely sandfly proof.	08/25
**Non-randomised Controlled Trial**													
Mondal 2010 Bangladesh (Mymensingh and Rajshahi districts) Non-randomized controlled trial [15]	The study was conducted from March 2008 to August 2009. 6844 HH from Putijana and 7968 HH from Deopara participated in the study Sand fly densities were measured at baseline and at 1, 12 and 18 months after the intervention.	✓			*Phlebotomus argentipes*	The results of the household interview surveys at 1 month after dipping showed that 94.4% of respondents in Putijana and 70.0% in Deopara perceived them to be protective against sand flies and other insects. The levels of acceptance dropped 6 months later to 84.4% in Putijana and 50.0% in Deopara as a decrease in efficacy against insect nuisance was perceived by the communities. Each household possessed on average 1.8 and 2.5 bed nets and 97.7% and 90.3% of existing bed nets were dipped in Putijana /Mymensingh and Deopara / Rajshahi, respectively.			Sandfly densities at different time points before and after net treatment showed that the dipping programme significantly reduced the sandfly density in the intervention areas compared to the control areas. The observed reduction by the intervention was about 60% for 18 months period.	Regarding the bioassays, the Abbot corrected sandfly mortality at 24 h after they had been exposed to the treated nets was high at 1 month and at 12 months after the dipping. However, thereafter, the mortality rate dropped but remained at about 80% threshold level even at 18 months after dipping across the two sites.	The chemical analysis showed that the average concentration of deltamethrin in the treated nets dropped over 18 months from 24 to 9.5 mg/m2.	The study did not measure the sandfly reduction beneath the impregnated nets, which the researchers assume is more marked than outside the nets, or in unprotected sentinel houses in intervention communities, measuring the mass effect of the impregnation programme on the sandfly population. The reduction in parasite transmission and subsequently in VL incidence by effective vector control was not measured in this study and it is still an open question.	14/18
**Community Intervention Trials**													
Mondal 2013 Bangladesh (Rajshahi–Godagari) Community intervention trial (+in-depth interview with each HH head) [17]	The intervention area was 36 villages in Deopara union comprising 2,512 households (11,426 inhabitants), the control area was the 36 villages from other 4 unions comprising 3,143 households (14,021 inhabitants) Intervention took place from February to March 2008. Follow up period: December 2009–January 2010	✓			*Leishmania donovani*	The use of impregnated bed-nets was very high (99.8%).		Before intervention, 69 VL cases were found (27/10,000 persons) in the study area. VL incidence in the intervention area (37.6/10,000 persons) was significantly higher than in the control area (18.5/10,000 persons). After intervention, VL incidence in intervention and control areas was 2.6 and 8.6 cases per 10,000 persons, respectively. During follow up, annual VL incidence declined in both areas, with a greater reduction in the intervention area (decrease of 35 cases/10,000 persons) than in the control area (decrease of 9.99/10,000). The effect of community-level intervention, measured by difference in-difference method, was 66.5%. By odds ratios the study found that 85.8% of the population in the intervention area was protected from VL by the intervention. The effect of the intervention in reducing VL-affected HHs in the intervention area compared with the control area was 70.5% by difference-in-difference analysis. By odds ratios, the estimation of the crude protection of HHs in the intervention area was 87% compared with those in the control areas.				The finding or this study are not consistent with those of Picado et al. [21], who found no additional protection by random village wise distribution of commercial insecticide–treated bed-nets compared with existing vector-control practice in India and Nepal. However, this discrepancy might be explained by the different delivery (commercial bed-net vs. existing bed-net impregnation) and coverage achieved (patchy village wise vs. all villages in the area) by the intervention.	12/18
Banjara 2015 Bangladesh, India and Nepal Community intervention trial (pre-post design) [30]	The study covered four villages in each of the three countries (number of HHs in study villages were 392 in Bangladesh, 2048 in India and 300 in Nepal). The study was conducted from June to august 2013. Baseline was at 2 weeks before intervention and follow up was done 4 and 24 weeks after the impregnation of bed nets in intervention and control villages.	✓			*Phlebotomus argentipes/Leishmania donovani*	During the study it was found out that HHs having untreated bednets were 97.2% in Bangladesh, 59.3% in India, and 89.3% in Nepal. The median number of bednets per household was two. Regular use of bednets by all family members of the household was 58.7% in Bangladesh, 23.2% in India and 67.7% in Nepal. The bednet impregnation rate was 82.1% in Bangladesh, 81.5% in India, and 99.8% in Nepal.			The reduction in sandfly density after 2 weeks of bednet impregnation was 86.5% and 32.6% in India and Nepal respectively as compared to baseline and the reduction after 4 weeks was 94.6% and 12.5% in India and Nepal respectively. There was no measurement in Bangladesh due to staff shortage. The reduction was significant in India and non-significant in Nepal for both measurements.			The non-significant results obtain in Nepal for reduction of vector population could be because in Nepal both intervention and control villages were covered additionally by indoor residual spraying with pyrethroid insecticides so that the impregnation of bednets did not provide an additional significant benefit. The main limitation of this study was the small sample size; however, it was possible to show in this exploratory study that it is feasible to conduct combined fever camps within the context of national control programs and that the potentials for detecting several fever diseases and organizing at the same time vector control are promising for the maintenance phase of the VL elimination initiative.	15/18
Das 2014 Nepal Crossover field trials (using each participant as their own control) [31]	The study used a total of 20 cattle sheds, 10 in each crossover trial, in the Bhathigachha village in the Morang district in Eastern Nepal The intervention trials were conducted from October to November 2013.		✓Trial 1: mesh sizes 156 holes/inch2 and 625 holes/inch2.Trial 2: LLIN & untreated nets, same mesh size		*Phlebotomus argentipes/Leishmania donovani*				At baseline, the cattle sheds selected for trial 1 and trial 2 (10 sheds per trial) had a median of 36 and 45.5 *P*. *argentipes* collected respectively. Fewer *P*. *argentipes* were captured inside the 625 mesh nets (n = 514 *P*. *argentipes* in net B) compared to 156 mesh nets (n = 561 in net A) in trial 1. In this trial it was observed that the use of 625 mesh size nets (B) reduced by 77% and 78% the number of female *P*. *argentipes* and total *P*. *argentipes* captured inside the nets respectively compared to 156 mesh size nets (A). In trial 2 fewer *P*. *argentipes* were collected inside the treated nets (n = 527 in net A) than inside untreated nets (n = 577 in net C). The results of this trial show that using a-cypermethrin treated nets (A) reduced by 77% and 61% the number of female and total *P*. *argentipes* collected inside the net respectively compared to untreated nets (C).	The mean *P*. *argentipes* mortality per LN was 98% and 94% for the two treated nets: net A with 200 mg/m2 and net B with 160 mg/m2 respectively. The laboratory sample of a 625 mesh net with 200 mg/m2 of alpha-cypermethrin had an average mortality of 97%.		The use of cattle sheds in the study may have precluded an estimate of the absolute barrier effect of the different types of net since cattle sheds also constitute one of the probable breeding and resting sites for *P*. *argentipes* and some of the sand flies captured inside the nets may have newly emerged from the substrate beneath the net. However, the researchers indicate that this is unlikely to be a major source of error and would not invalidate the main findings of the study.	15/18
Kumar 2015 India (Maldah and Burdwan districts) Community intervention trial (Pre-post design) [32]	The study was conducted in two villages each in Maldah (Tapsahor and Nimua) and Burdwan (Barapolason tola and Mahishpur) districts The intervention took place in April-May and November-December, 2010. The follow up period was done from March 2011 to December 2012.		✓ PermaNet 2.0		*Phlebotomus spp*. *And Sergentomyia spp*					Regarding the bioassay results, there was 60% mortality in test against LN PermaNet 2.0 within the 3 min of exposure time. The test against DDT on wall illustrated 50% mortality of sandflies with 30 min time of exposure in the study area. LN PermaNet 2.0 showed higher efficacy by having higher mortality with low does and exposure time compared with DDT.		The sample size of the study is relatively small and a larger study in needed covering the whole State with more number of *P*. *argentipes* to monitor the resistance in the vector species for the effective kala-azar control.	11/18
Karakus 2016 Turkey [Not from the South Asian regional initiative] Intervention study [14]	The study was done at Kızıllar village. For the intervention the bed nets were distributed in may 2013. Follow up was 6 months after intervention.		✓ Olyset Plus nets		*Phlebotomus spp*. *And Sergentomyia spp*.				The groups according to bed net set up and conditions are as follow: -A1 represents participants that prefer to sleep outdoors over the 6-month transmission period and do not take down their bed nets -A2 represents the participants that prefer to sleep outdoors but under bed nets that are not exposed to sunlight and rain as bed nets are dismantled after each use and put away -A3 represents the participants that prefer to sleep indoors. This group was set up indoors and did not put the net away after use for the whole study period. -B1 represents the participants that prefer to sleep indoors and pack up and store their bed nets after use during the daytime. -Control group 1 (C1) was generated using the retrieved bed nets from families which were used following the instructions on the flyer of the product. -Control group 2 (C2) were left in their original packaging, which was opened only at the start of the bio-efficacy test.	Mortality rate was found to be 100% for five groups (A2, A3, B1, C1, C2) whereas group A1 was found to have a lower mortality rate (44.4%) by the end of 24 h. The control group, which used untreated bed nets, 3 out of 120 specimens died after 24 h. A1 and C1 groups were found to have a similar and lower knock down effect after 1 h.	Indoor bed net groups (C2 and A3) showed a full knock down effect on the tested sand flies by the end of 1 h. Similarly, a significant knock down effect was observed on group C2 with 100% by the end of first 30 min. Results of gas chromatography analysis showed no significant decrease on PBO and permethrin contents.	The researchers could not use a data logger on each set up site to record the micro-changes in the temperature, which could have provided good data for comparison of the bed net groups. Another limitation is that they could not do the cone test experiments using sandflies collected from the study area because of the unknown status of insecticide resistance.	13/18
Singh 2016 India (Bihar) Community intervention study (pre-post design) [18]	The study was conducted in four villages Hulashchak, Gonpura, Duparchak, Nagawa of Patna district state of Bihar. In total 625 HHs were part of the study. The intervention and follow up periods were from August 2007 to November 2008		✓ PermaNet 156 mesh/inch2, PermaNet 196 mesh/inch2 and PermaNet 196 mesh/inch2 + 75 cm border of fine cloth		*Phlebotomus argentipes*	All the three types LLINs were accepted by communities, as these distributed free of cost. Household survey revealed that PermaNet 156 mesh/inch^2^ mesh was preferred by 100%, PermaNet 196 mesh/inch^2^ by 91.7%, while PermaNet 196 mesh/inch^2^+75 cm border was the least preferred 54.2% as this net does not provide air circulation during sleep at night.			The reduction of *P*. *argentipes* population observed in the first month of post intervention of LLINs were as follows: 100 in Hulashchak, 87.3 in Gonpura and 85.55% in village Duparchak. The minimum reduction of *P*. *argentipes* population were 36.66 and 35.55% in 9th and 13th month, while a sharp increase was noticed in 12th month in Hulashchak. In Gonpura sharp decline recorded throughout the year between 73.41 to 100%, minimum in 12^th^ month 72.41% post intervention. Dhuparchak where mesh size was196 + 75 cm of border found maximum decline in P. argentipes population throughout the year 85–100%, in 8th month of treatment only 71.1% decline was recorded. In the entire intervention period, maximum reduction 93.67% was observed in Duparchak PermaNet 196 mesh/inch^2^ + 75 cm border followed by Gonpura PermaNet 196 mesh/inch^2^ 91.90% and 74.29% in Hulashchak PermaNet 156 mesh/inch^2^, when compared to control all the LLINs were significant; while compare to between the study arms there is no significant p values. The significant reduction in gravid *P*. *argentipes* 71.87, 87.92 and 91.27% were also noted in treated villages Hulashchak, Gonpura and Duparchak, respectively.			Not specified	10/18
Dinesh 2008 India (Bihar) Intervention study (with purposively selected households) [33]	The study was conducted in the state of Bihar (India). Three hamlets were selected: Gulmehiya Bagh in Patna district, and Rasoolpur and Majlishpur, both located in Vaishali district. With a total of 48 HHs. The baseline was done before the intervention (week 0) and follow up was 3, 6 and 9 weeks after installing the nets (April–June 2006).		✓PermaNet–PT Olyset–OT and untreated nets (PermaNet Control–PC, local polyester untreated nets–LC).		*Phlebotomus argentipes and Sergentomyia* *Spp*.*/ Leishmania donovani*				Compared with LC, there is significant reductions in males of *P*. *argentipes* with OT and PT. These reductions are not significant for females of *P*. *argentipes*. For S*ergentomyia*, only OT results in a significant reduction in males and females. This reduction is mainly due to a reduced number of unfed *Sergentomyia*. OT results in a significant reduction of the pooled *P*. *argentipes* and *Sergentomyia*. CDC light trap collections performed better than aspirator collection for all the ‘groups’ except for fed females of *P*. *argentipes* and *Sergentomyia* for which aspirator collection in the morning was more effective. Furthermore, no significant differences were observed between the types of houses.			The design of the study could have affected the results since the study assumed that most sandflies are breeding inside the houses and the presence of treated nets inside a single house would progressively reduce the sandfly population in that house during the reproduction season. As such decline was not observed, they postulate that most of the sandflies are breeding outdoors. Another limitation is that a possible mass effect, as well as their impact on morbidity, mortality and personnel protection was not measured in this study. Therefore, LLINs should certainly not yet be dismissed as a potential tool in the fight of kala-azar based solely on this data.	14/18
Courtenay 2007 Brazil [Not from the South Asian regional initiative] Intervention Study (Crossover field study) [12]	The study was conducted in the community of Pingo d ‘ Agua, municipality of Salvaterra, Marajó Island, Pará State, Brazil. In total, 4 houses were enrolled in the study The study was conducted between July and August 2003 (dry season)		✓ ITN (deltamethrin impregnated)		*Lutzomyia longipalpis/ Leishmania infantum*				A total of 1284 female sandflies were collected inside houses during the 30 trapping night crossover study. Adjusting for house and night effects, the mean absolute number of female sandflies captured per night in ITN houses was 14.4, which was not dissimilar to the mean of 16.9 collected in houses with untreated nets. The mean number collected per night under treated nets was 1.3, which was significantly fewer than the mean of 2.6 collected under untreated nets. These data indicate that the insecticide increased the barrier effect of untreated nets by an average of 39.2%. The insecticide also reduced the percentage of (collected) sandflies landing under nets from 71.4% in untreated to 14.5% in treated households, representing a reduction of 79.7%. The 24-hour mortality rate of all sandflies collected under ITNs was 97.7%, compared with 0% under untreated nets. Mortality rates for the sandflies on the surface of the net and landing on collectors under ITNs were 32/32 and 4/5 compared with 0/23 and 0/56 under untreated nets, respectively.		The absolute number of sandflies landing exterior to ITNs was reduced despite a similar abundance of sandflies in rooms with the two respective treatments. The rate of 24-h mortality amongst sandflies collected exterior to ITNs was 67.7%, compared with 0.4% of those collected exterior to untreated nets. Within ITN houses, the 24-h lethality effect of the insecticide was greater inside than outside the net, and greater amongst flies alighting on the walls than landing outside the nets.	Sample size could have limited the results. A potential predominant limiting factor for ITN efficacy in this study region is a social one: bedtimes were late relative to peak sandfly activity times. In this case, use of additional protective measures such as repellent DEET before children’s bedtimes would be advantageous, although it is unlikely to be perceived as affordable.	13/18
Elnaiem 1999 Sudan [Not from the South Asian regional initiative] Intervention study (+Sociological surveys) [11]	The study was undertaken in two villages (Bellow and Ai-Elgamel) and Dinder National Park (DNP). 441 people answered the survey. The study period was June 1995.		✓ Bed nets impregnated with the pyrethroid insecticide lambda-cyhalothrin		*Phlebotomus orientalis*		For different sex and age groups of the people living in Bellow and Ai-Elgamel villages, the daily potential duration of exposure the users of impregnated bednet have to the risk of *P*. *orientalis* biting, was taken as the time after sunset until bed-time, the likely start of bednet use. The majority of people were found to have < 1 h (39.5%) or 1–2 h (49.9%) of potential exposure to sandfly bites. Nearly 11% of people were found to be exposed to the risk of sandfly bites for more than 2 h before bed-time. The duration of exposure to the risk of sandfly bites was associated with gender as well as the age group. Females had significantly less exposure time than males. Children <5 years experienced the least exposure, with most individuals (92.9%) having <1 h exposure. For the age group 6–15 years, potential exposure time was mostly 0–1 h (52.7%) with nearly as many having 1-2h (45.5%), and a negligible proportion having >2 h exposure. Two-thirds (66.8%) of people >16 years old were found to be exposed to risks of sandfly bites for 1–2 h, with lower proportions having <1h (14.8%) or >2 h (3.3%) duration of exposure risk before going to bed.				The mortality rate of *P*. *orientalis* was 100% (n = 4310 females) within 1 h post-exposure for 30s to cage netting treated with lambda-cyhalothrin. For the untreated controls (n = 4310 females) the survival rate was 100% for 24 h. Sandfly biting was zero for persons using the impregnated bednets and significantly reduced for persons staying under untreated bednets (6.92+/-2.71 bites/man/night), whereas persons without bednets experienced 32.0+/-8.3 bites/man/night.	Not specified	10/18
Kumar 2017 India Comparison-based intervention study [34]	The study was undertaken in the Samastipur district, 4 villages of the district were selected for the intervention (Mirzapur, Sahnitola, Nifsy and Bisanpur). In total 400 HHs were targeted for the study. The study period was from October 2014 to October 2015		✓ Perma Net 3.0	IRS+ITN	*Phlebotomus argentipes/ Leishmania donovani*	All respondents facilitated with ITNs, confirmed the proper usage of provided bed nets as well as the continuous good physical conditions of the nets. Almost the only reported side effect was unpleasant smell, particularly in the 2 arms that included IRS and IRS+ITN. The perception of added benefits (mainly reduction in nuisance of insects) was highest in villages where ITNs were involved. Overall satisfaction was achieved in the villages involving ITNs as compared to the village with IRS as single intervention with 87% acceptability.			The lowest numbers/ proportions of sand flies were collected from villages with the combined approach (IRS plus ITN) as compared to single intervention sites (either IRS or ITN only) or the control site. The reduction of insecticidal content of IRS was faster and more pronounced (exhibiting corrected mortality rate as 52.38%, 58.33%, 45.45% & 50.00%) as compared to ITN (with corrected mortality rate as 84.44%, 82.50%, 77.78% & 83.33%) over the period of 13 months since intervention. The Monthly observation of percent reduction (%RI) of sand fly density due to intervention establishes the highest % RI (93.59–100%) at the sites with IRS+ITN as compared to either at the control site (with 0% reduction) or with single intervention of IRS (with 4.29–86.77%) or with ITN (60.18–97.01%). At the site with the combined treatment of IRS+ITN, no re-emergence of sand flies was recorded till 13 months following the intervention.			The expected outcome at the villages of Sahnitola (for intervention with ITN only) was insignificantly hampered due to poor literacy rate i.e., 28% only.	15/18
**Observational Studies**													
Chowdhury 2019 Bangladesh (Fulbaria) Retrospective cohort study [16]	Three interventions with a total of: 8142 (HH in intervention) (37050 pop.), 7729 (HH in control) (34930 pop.) Follow up period for the three interventions was: 2004 (period of observation 2001–2007) 2006 (period of observation 2003–2009) 2008 (period of observation 2005–2011) Three year follow up period for the three interventions.	✓	✓		*Phlebotomus argentipes/Leishmania donovani*	Of the intervention HHs more than 92.2% had at least one bed net in their house at the time of the household survey. Among those, 80.1% were ITNs, either self-impregnated with K-O TAB 1-2-3 or LLIN, the others were non-impregnated commercial nets.		A total of 1011 VL cases were recorded in the three years previous to the interventions (534 cases in the intervention areas; 144.13/10,000/year) (477 cases in control area; 136.59/10,000/year). After the three-year intervention period a total of 555 VL cases were identified (178 cases in the intervention area; 48.04/10,000/year) (377 cases in control areas; 107.95/10,000/year). The effect of the intervention was strongly significant. The estimated reduction of VL incidence rate by the intervention was 46.80%. Male were more affected by VL than females. 75% of the VL incidence occurred in the age range of 2 to 30 years.				The study is non-experimental in nature, there could be other factors that explain the trend in incidence rates (IRs) in the cohorts, such as e.g. a more intense screen-and-treat as the baseline IR were of the highest in the region, and communities might have been targeted preferentially by the programme. The comparison is a one-to-one comparison of one cohort compared to another and given the erratic behaviour of VL in small areas, the lack of replicates limits the robustness of the findings. In the given context of very low case incidence, the organization of randomized trials was deemed not feasible, which lead to possible biased results.	15/18
Ritmeijer 2007 Sudan [Not from the South Asian regional initiative] Cohort study [10]	357064 insecticide-treated bednets (ITN) were distributed to 155 affected villages in southern Gedaref state. The intervention was developed from October 1998 to March 2001. The baseline and follow up period was measured from March 1996 to June 2002.		✓		*Phlebotomus orientalis*	376667 beneficiaries resulted from the ITNs distribution in the 155 villages of the study, resulting in a 94.8% coverage. In the three villages of Afrosh Tobak, Berber Fugera and Um Bileil, of the 503 nets received by the 91 households interviewed, 328 (65.2%) were still present in the receiving family. Of the missing nets, 77 (15.3%) were reported to be with family members elsewhere. 37 nets (7.3%) were reportedly sold. Of the 328 bednets still present in the families, 115 (35.1%) were in good condition, 104 (31.7%) were slightly damaged, 109 (33.2%) were in bad condition. Bednets were most frequently used during the rainy season. During April and May, which are the hottest months of the dry season and the early part of the VL transmission season, bednet use was <10%. After the first rains in June, people shifted to sleeping indoors, and bednet use increased to 55%. Sleeping behaviours tend to reduce the protective effect of ITNs on children and the different season also play a role in the use of ITNs by the HH members.		The study found that the number of cases reported by village and month was significantly reduced following ITN provision at all 4-month time points up to 20 months post-intervention. The greatest effect was detected 17–20 months post-intervention, with the number of cases on average reduced by 59%. The predicted number of cases in each village and each month in the absence of bednets from June 1999 to January 2001 was 3863, which compares of an observed number during that time of 2803. Therefore, bednet intervention could have reduced the number of cases up to January 2001 by 1060, with a calculated protective effect of 27.4%				In the study the number of cases potentially reduced by the bednets after the last distribution date cannot be estimated as there were no remaining villages without bednets to make the comparison. The figures on VL cases must be treated with caution, as the estimates of apparent effectiveness may have been influenced by the non-random sequence of net allocation to villages. There are a number of reasons why the estimate of the epidemiological impact of ITN provision may be seriously inaccurate. First, incidence figures used in the epidemiological analysis were derived from MSF clinics, and therefore only represent reported VL cases, and not true incidence. Second, increased awareness of VL after the health education activities may have resulted in improved health-seeking behaviour for VL, thus potentially biasing post-distribution incidence data. Third, it is clear that the order with which villages were provided with ITN during the control campaign was significantly biased towards those with more reported cases. The non-significance of the protective effect after 20 months post-distribution is probably a sample size issue, as there are fewer comparisons to make after 20 months.	13/18

VL, Visceral Leishmaniasis; cRCT, cluster randomized controlled trial; ITN, insecticidetreated net; Pop., population; RCT, randomized controlled trial; HH, household; FC, fever camp; CI, confidence interval; NKTA, no kala-azar transmission activity; DWL = insecticide impregnated durable wall lining; LN/LLIN, Longlasting insecticidal net; KD, Knock down effect; HPLC, high performance liquid chromatography; IRS, indoor residual spraying; EVM, environmental modification; KOTAB, insecticide K-O TAB 1-2-3; OUT, insecticide spraying in potential breeding sites outside of house using chlorpyrifos 20EC; PBO, piperonyl butoxide; PT, Permanent; OT, Olyset; LC, local polyester untreated nets; PC, PermaNet Control; DEET, N,N-Diethyl-meta-toluamide; DNP, Dinder National Park; RI, reduction due to intervention; IR, incidence rate; MSF, Médecins Sans Frontières.

### Description of included studies

#### Time and geographical clustering of studies

All included articles were published between 1995 and 2019. Articles were published by different groups of authors and focusing mostly (20 articles) on South Asia (Bangladesh, India and Nepal). Ritmeijer, Davies [10] and Elnaiem, Elnahas [11] focused on Africa (Sudan), Courtenay, Gillingwater [12] and Alexander, Bruce [13] on Latin America (Brazil and Colombia, respectively) and Karakus, Kasap [14] on Turkey.

#### Methods/designs applied by the studies

The 25 studies selected for the review included both interventional (23 studies) and observational designs (two studies) (Table 1). Among the 23 interventional studies, 11 are cRCTs (including one multi-country, two paired and one community RT), two are RCTs, one is a non-randomised controlled trial [15], and 10 are community interventions trials including comparison-based intervention studies, pre-post designs and cross-over field trials. Among the two observational studies, one followed a cohort design (10) and the other followed a retrospective cohort design [16]. Surveys and in-depth interviews were also part of few studies [11,17,18].

#### Types of interventions

The interventions evaluated or assessed in the studies included impregnation of existing bed nets with a slow-release insecticide tablet/KO Tab 1-2-3 (eight studies), long-lasting insecticidal nets, Long-lasting or long lasting insecticidal or insecticide-treated or insecticide impregnated nets or bed-net (18 studies) including PermaNet, PermaNet 2.0, PermaNet nets with different mesh sizes (156 holes/in^2^, 196 holes/in^2^, 625 holes/in^2^), polyester, resin coating, containing deltamethrin, PermaNet 3.0, Olyset and Olyset Plus (Table 1). Interventions combining multiple vector control methods (Indoor residual spraying (IRS)+long-lasting insecticidal net (LLIN), IRS+KOTAB, LLIN+outdoor spraying) were also assessed in 2 studies.

#### Types of outcome measures

Studies involved outcome measures focusing on sandfly vectors (entomological) and human hosts parameters (Table 1). There were several entomological outcomes in the included articles, in some cases the studies evaluated more than one entomological outcome. The measured outcomes included sandfly density (17 studies), sandfly mortality (eight studies), and Insecticide residue on ITNs, sandfly landing and knock down of sandflies (six studies). Among the human outcomes there were perception, acceptance, access, utilization, maintenance and satisfaction regarding ITNs (10 studies), feeding on human blood and sandfly biting (two studies), and VL incidence, *Leishmania donovani* (*L*. *donovani*) infection, and seroconversion (four studies). Like the entomological outcomes, some studies also measured more than one human outcome.

### Quality analysis

There was a high variability of appropriateness of study designs, study sizes and consistent limitations in finding sufficient number of VL cases in low incidence settings. Variations were also observed for the comparability between the intervention and control arms, the way vector density was measured, and cases were identified, and also how potential confounders were taken care of. As a result, the quality assessment scores performed using the CONSORT checklist varied from 10/18 to 21/25 (Table 1). No study was excluded because of a low score.

## Analysis of results

### Entomological outcomes

#### Sandfly density

A study from Bangladesh testing both KO Tab 1-2-3 (KOTAB) impregnated bed nets and LLIN showed that LLIN was effective from second follow-up (at 4 months) onwards through to the end of the 22 months follow up period [28]. The adjusted model for LLIN showed that the vector density (males plus females) reduction was 9% to 78% and the rate ratio was between 0.91 and 0.32 for two years. In the KOTAB arm, a significant sand fly density (males plus females) reduction was observed only at the third (at 5 months), sixth (14 months), seventh (15 months) and eighth (18 months) follow-up measurements. For the other combined intervention arms: The combination of IRS with LLIN (IRS+LLIN) or with KOTAB (IRS+KOTAB) and with outdoor spray (IRS+OUT) showed statistically significant *Phlebotomus argentipes* (*P*. *argentipes*) (males plus females) reductions almost all through the study period. The adjusted model showed that the effects of IRS+LLIN and IRS+KOTAB on sandfly density reduction (males plus females) were 23.0% to 85.0% and 16.0% to 86.0%, respectively. The combination of LLIN with outdoor spray (LLIN+OUT) was found to be effective throughout the study period at a highly significant level. The reduction of *P*. *argentipes* sand fly density (males plus females) in this case was 26.0% to 86.0%.

In another study from Bangladesh, the mean reduction of female *P*. *argentipes* counts in the ITN arm ranged from −6.10 (95% CI: −3.04, −9.19) to 0.81 (95% CI: 0.01, 1.60). The adjusted intervention effect of ITN was statistically significant on reduction of the incidence rate of female *P*. *argentipes* sandfly count up to 3 months post-intervention [19]. In another study, a dipping programme significantly reduced the sandfly density (males plus females) in the intervention areas compared to the control areas. The observed reduction by the intervention was about 60% for 18 months period [15]. The reduction in sandfly density (males plus females) after 2 weeks of bednet impregnation (KOTAB 123) was 86.5% and 32.6% in India and Nepal, respectively as compared to baseline and the reduction after 4 weeks was 94.6% and 12.5% in India and Nepal, respectively [30].

In a multi-country cRCT in Bangladesh, Nepal and India, bed nets impregnation with KO Tab 1-2-3 reduced female sand fly density through to 9 months [20]. In India and Nepal, in a paired cRCT, indoor density of *P*. *argentipes* (males plus females) was reduced by 25% in the study clusters that used treated nets compared with control clusters [21]. The effect was not significantly different in the two countries. The analyses carried out on *P*. *argentipes* females also found a significant reduction in density of 11.6% [26]. For LLIN in Nepal, vector density (males plus females) dropped from 7.9 to 0.9 per house per night by LT collections and from 1.8 to 0.5 per house per morning aspirator collections in three follow-up surveys after intervention [24].

When the net effect of individual intervention in reducing sandfly count (males plus females) was estimated, the following was found: a 72.4% reduction for IRS, a 42.0% reduction for EVM and a 43.7% reduction for LLIN (PermaNet) [25]. Both IRS and ITNs were associated with a 70–80% decrease in the density of *P*. *argentipes* 4 to 5 months after the intervention (males plus females). Households (HHs) in the ITN arm continued to show significantly lower vector density compared with the control arm, 11 months after intervention (60% lower densities) [27]. In a study from India, the highest monthly percent reduction (% RI) of sandfly density (males plus females) was observed at the sites with IRS and ITN (93.59–100%) as compared to either at the control site (with 0% reduction) or with single intervention of IRS (with 4.29–86.77%) or with ITN (PermaNet 3.0) (60.18–97.01%). At the site with the combined treatment of IRS and ITN, no re-emergence of sand flies was recorded till 13 months following the intervention [34].

While compared between two mesh sizes in a cross-over design [31], fewer *P*. *argentipes* (males plus females) were captured inside the 625 (holes/inch^2^) mesh nets (n = 514 *P*. *argentipes* in net B) compared to 156 (holes/inch2) mesh nets (n = 561 in net A). In this trial it was observed that the use of 625 mesh size nets reduced by 77% and 78% the number of female *P*. *argentipes* and total *P*. *argentipes* captured inside the nets, respectively, compared to 156 mesh size nets (A). When compared with untreated nets (n = 577), fewer *P*. *argentipes* were collected inside the treated nets (n = 527). Using a-cypermethrin treated nets reduced by 77% and 61% the number of female and total *P*. *argentipes* collected inside the net, respectively, compared to untreated nets.

In another study in Bihar, India, three different mesh sizes of PermaNet LLINs were evaluated—156 mesh/inch^2^, 196 mesh/inch^2^ and 196 mesh/inch^2^ + 75 cm border of fine cloth [18]. LLINs were distributed to cover all the members of the family. The reduction of *P*. *argentipes* population (males plus females) observed in the first month of post intervention of LLINs were as follows: 100% in Hulashchak, 87.3% in Gonpura and 85.55% in village Duparchak. The minimum reduction of *P*. *argentipes* population were 36.66 and 35.55% in 9th and 13th month, while a sharp increase was noticed in 12th month in Hulashchak. In Gonpura sharp decline recorded throughout the year between 73.41 to 100%, minimum in 12^th^ month 72.41% post intervention. Dhuparchak where mesh size was196 + 75 cm of border found maximum decline in *P*. *argentipes* population throughout the year 85–100%, in 8th month of treatment only 71.1% decline was recorded. In the entire intervention period, maximum reduction 93.67% was observed in Duparchak PermaNet 196 mesh/inch^2^ + 75 cm border followed by Gonpura PermaNet 196 mesh/inch^2^ 91.90% and 74.29% in Hulashchak PermaNet 156 mesh/inch^2^, when compared to control all the LLINs were significant; while compared to between the study arms there was no significant p values. The significant reduction in gravid *P*. *argentipes* by 71.87%, 87.92% and 91.27% were also noted in treated villages Hulashchak, Gonpura and Duparchak, respectively.

A cross-over study from Brazil testing deltamethrin impregnated bed nets against *Lutzomyia longipalpis*, has shown that the mean number of sandflies (males plus females) collected per night under treated nets was 1.3, which was significantly fewer than the mean of 2.6 collected under untreated nets. These data indicate that the insecticide increased the barrier effect of untreated nets by an average of 39.2%. The insecticide also reduced the percentage of (collected) sandflies landing under nets from 71.4% in untreated to 14.5% in treated households, representing a reduction of 79.7% [12]. Another study from the Latin America (Colombia) has shown that deltamethrin impregnated curtain did not significantly reduce the mean numbers of sandflies collected per man-hour in rooms [13].

#### Sandfly mortality

A wide range of mortality rates for sandfly have been found in studies at varying intervals with different interventions. A dose-response relationship has been observed with higher mortality rates in earlier follow-ups.

The Abbott’s corrected sand fly (males plus females) mortality rate (inside of test containers) [mean and 95%CI] at 1, 3, 9 and 12 months after bed nets impregnation with KO Tab 1-2-3 was respectively 75% (71%-79%), 67% (64%-74%), 63% (57%-68%) and 49% (43%-55%) [20]. In another study, for the same intervention, mortality (inside of test containers) of *P*. *argentipes* sandfly (males plus females) was 51.7% at the 12 months follow up [19]. In an earlier investigation in Bangladesh, sandfly (males plus females) mortality (inside of test containers) at 24 h after they had been exposed to the treated nets was high at 1 month and at 12 months after the dipping. However, thereafter, the mortality rate dropped but remained at about 80% threshold level even at 18 months after dipping across the two districts–Mymensingh and Rajshahi [15]. In a longer follow-up up to 20 months, Chowdhury, Faria [28] have shown sandflies (males plus females) mortality (inside of test containers) on K-O TAB 1-2-3 impregnated nets dropped from 88.37% at 3 months to 69.12% at 20 months after use. In the same study, morality for LLIN was also tested and found to remain as high as 83% at 20 months of use. In a cross-over design in Nepal involving two mesh sizes, the mean *P*. *argentipes* (males plus females) mortality (inside test containers) per LLIN was 98% and 94% for the two treated nets with 200 mg/m^2^ and 160 mg/m^2^, respectively [31]. The laboratory sample of a 625 mesh net with 200 mg/m^2^ of alpha-cypermethrin had an average mortality (inside test containers) of 97%.

In a study in Brazil, the 24-hour mortality rate (outside of test containers) of all sandflies (males plus females) collected under ITNs was 97.7%, compared with 0% under untreated nets. Mortality rates (outside of test containers) for the sandflies (males plus females) on the surface of the net and landing on collectors under ITNs were 32/32 and 4/5 compared with 0/23 and 0/56 under untreated nets, respectively [12]. In Colombia, *Lu*.*youngi* showed significant knockdown within 1 h (13.2%) and mortality after 24 h (43.4%) of exposure to impregnated curtains [13]. *Lu*. *Youngi* collected resting on impregnated bednets showed a mean knockdown rate of 7.1% after 1h and mortality of 39.7% after 24h, significantly higher (p < .05) than the rates for those caught resting on untreated walls of houses without impregnated bednets or curtains. In a study in Turkey, testing different insecticide-treated bed net set ups including indoor and outdoor, and taking down and not taking down the nets, sandflies (males plus females) mortality rate (inside test containers) was found to be 100% for all the groups, whereas the group representing participants who preferred to sleep outdoors over the 6-month transmission period and did not take down their bed nets had a lower mortality rate (inside test containers) (44.4%) by the end of 24 h. The control group, which used untreated bed nets, 3 out of 120 specimens died after 24h [14]. In Sudan, the mortality rate (inside test containers) of *P*. *orientalis* (females) was 100% (n = 4310 females) within 1 h post-exposure for 30s to cage netting treated with lambda-cyhalothrin. For the untreated controls (n = 4310 females) the survival rate (inside test containers) was 100% for 24 h [11].

#### Insecticide residue on ITNs, sandfly landing and knock down (KD) of sandflies

After 22 months of use, 36.6% of nets (PermaNet 2.0) in India and 22.2% in Nepal were still intact [22]. The chemical analysis showed that the average concentration of deltamethrin in the treated nets dropped over 18 months from 24 to 9.5 mg/m^2^ [15]. When compared, the reduction of insecticidal content of IRS was faster and more pronounced (exhibiting corrected sandflies (males plus females) mortality rate as 52.38%, 58.33%, 45.45% & 50.00%) as compared to ITN (PermaNet 3.0) (with corrected sandflies (males plus females) mortality rate as 84.44%, 82.50%, 77.78% & 83.33%) over the period of 13 months since intervention. [34].

Use of deltamethrin impregnated net in a Brazilian community showed reduction in the percentage of (collected) sandflies (males plus females) landing under nets from 71.4% in untreated to 14.5% in treated households, representing a reduction of 79.7% [12]. The absolute number of sandflies (males plus females) landing exterior to ITNs was reduced despite a similar abundance of sandflies in rooms with the two respective treatments. The rate of 24-h mortality (outside of test containers) amongst sandflies (males plus females) collected exterior to ITNs was 67.7%, compared with 0.4% of those collected exterior to untreated nets (outside of test containers). Within ITN houses, the 24-h lethality effect of the insecticide was greater inside than outside the net, and greater amongst flies (males plus females) alighting on the walls than landing outside the nets.

The mean sandflies (males plus females) KD per swatch ranged from 88.5–99.0% after 12 months and 87.6–99.0% after 24 months, as observed in a multicountry study involving India and Nepal [22]. The mean mortality per swatch ranged from 91.0–100.0% after 12 months and 87.6–100.0% after 24 months. Bioassays were performed to test residual efficacy of DDT in the intervened households; regarding these results, there was 60% sandflies (males plus females) mortality (inside test containers) in test against LN PermaNet 2.0 within the 3 min of exposure time. The test against DDT on wall illustrated 50% mortality of sandflies (males plus females) with 30 min time of exposure in the study area. LN PermaNet 2.0 showed higher efficacy by having higher sandflies (males plus females) mortality with low does and exposure time compared with DDT [32].

### Human outcomes

#### Perception, acceptance, access, utilization, maintenance and satisfaction regarding ITNs

A study from Nepal showed that 72% of the respondents perceived a reduction of sandflies after bed net impregnation with insecticide [29]. Transient itching was reported by 4.8% of HHs with ITN [20]. Unpleasant smell was reported in 3.2% of ITN houses. After 22 months of use, 36.6% of nets in India and 22.2% in Nepal were still found to be intact [22]. The results of the household interview surveys at 1 month after dipping with KO Tab 1-2-3 in Bangladesh showed that 94.4% of respondents in Putijana and 70.0% in Deopara perceived them to be protective against sand flies and other insects [15]. The levels of acceptance dropped 6 months later to 84.4% in Putijana and 50.0% in Deopara as a decrease in efficacy against insect nuisance was perceived by the communities. In another study in Bangladesh, the use of impregnated bed-nets was found to be very high (99.8%) [17].

In a multi-country community intervention trial involving Bangladesh, India and Nepal, the bednet impregnation rate was 82.1% in Bangladesh, 81.5% in India, and 99.8% in Nepal [30]. When PermaNets were distributed as a part of intervention in Bihar, India, all the three types LLINs were accepted by communities, as these were distributed free of cost. Household survey revealed that PermaNet 156 mesh/inch2 mesh was preferred by 100%, PermaNet 196 mesh/inch^2^ by 91.7%, while PermaNet 196 mesh/inch^2^ and 75 cm border was the least preferred 54.2% as this net did not provide air circulation during sleep at night [18]. In another study from India, were IRS and ITN were evaluated in combination, all respondents facilitated with ITNs, confirmed the proper usage of provided bed nets as well as the continuous good physical conditions of the nets. Almost the only reported side effect was unpleasant smell, particularly in the 2 arms that included IRS and IRS with ITN. The perception of added benefits (mainly reduction in nuisance of insects) was highest in villages where ITNs were involved [18]. In Bangladesh, of the intervention HHs more than 92.2% had at least one bed net in their house at the time of the household survey. Among those, 80.1% were ITNs, either self-impregnated with K-O TAB 1-2-3 or LLIN, the others were non-impregnated commercial nets [16].

In Sudan, bednets were most frequently used during the rainy season. During April and May, which are the hottest months of the dry season and the early part of the VL transmission season, bednet use was <10%. After the first rains in June, people shifted to sleeping indoors, and bednet use increased to 55%. Sleeping behaviours tend to reduce the protective effect of ITNs on children and the different season also play a role in the use of ITNs by the HH members [10]. Overall satisfaction was achieved in Indian villages involving ITNs as compared to the village with IRS as single intervention with 87% acceptability [34].

#### Feeding on human blood and sandfly biting

In a study in Nepal and India, for *P*. *argentipes* the geometric mean of ELISA OD was used to evaluate exposure of sandflies species to human individuals in the study, the results showed that the exposure to the vector was, on average, 12% reduced at 12 months and 9% at 24 months in the intervention group compared to control group [23]. Similar results were obtained for *P*. *papatasi*: 11% and 9% reduction in LN group at 12 and 24 months respectively, the study evaluated *P*. *papatasi* but it has not been incriminated as a vector of VL. In addition, the percentages of positive samples for *P*. *argentipes* ELISA were reduced from 63.2% to 43.5% and from 47.1% to 43.6% in the intervention and control groups respectively over 24 months. For *P*. *papatasi*, the percentage of positive ELISA samples was not altered after 24 months in the control clusters (17%) but was reduced from 32.6% to 21.8% in the clusters using LN. The study from Sudan showed that sandfly biting was zero for persons using the impregnated bednets (bed nets impregnated with the pyrethroid insecticide lambda-cyhalothrin) and significantly reduced for persons staying under untreated bednets (6.92+/-2.71 bites/man/night), whereas persons without bednets experienced 32.0+/-8.3 bites/man/night [11]. For different sex and age groups of the people living in Bellow and Ai-Elgamel villages, the daily potential duration of exposure the users of impregnated bednet have to the risk of *P*. *orientalis* biting, was taken as the time after sunset until bed-time, the likely start of bednet use. The majority of people were found to have < 1 h (39.5%) or 1–2 h (49.9%) of potential exposure to sandfly bites. Nearly 11% of people were found to be exposed to the risk of sandfly bites for more than 2 h before bed-time. In another study in Latin America, in Colombia, the proportion of Lu. youngi females biting was significantly lower in rooms protected by curtains (0.48 v 0.65, p<0.05) but not for Lu.lichyi or Lu. columbiana, although they appeared to less predisposed to bite after passing through impregnated curtains [13].

The duration of exposure to the risk of sandfly bites was associated with gender as well as the age group. Females had significantly less exposure time than males. Children <5 years experienced the least exposure, with most individuals (92.9%) having <1 h exposure. For the age group 6–15 years, potential exposure time was mostly 0–1 h (52.7%) with nearly as many having 1-2h (45.5%), and a negligible proportion having >2 h exposure. Two-thirds (66.8%) of people >16 years old were found to be exposed to risks of sandfly bites for 1–2 h, with lower proportions having <1h (14.8%) or >2 h (3.3%) duration of exposure risk before going to bed.[11]

#### VL incidence, L. donovani infection, and seroconversion

From the selected studies, only two considered the effects of ITNs/LLINs on VL incidence [16,17]. After intervention with impregnation of bed nets with KO Tab 1-2-3 in Rajshahi, Bangladesh, VL incidence in intervention and control areas was 2.6 and 8.6 cases per 10,000 persons, respectively [17]. During follow up, annual VL incidence declined in both areas, with a greater reduction in the intervention area (decrease of 35 cases/10,000 persons) than in the control area (decrease of 9.99/10,000). The effect of the intervention in reducing VL-affected HHs in the intervention area compared with the control area was 70.5% by difference-in-difference analysis. By odds ratios, the estimation of the crude protection of HHs in the intervention area was 87% compared with those in the control areas. In a retrospective cohort study in Fulbaria, Bangladesh [16], where the communities had been benefited previously from a LLIN or K-O TAB 1-2-3 distribution in 1 of three distinct studies, after the three-year intervention period a total of 555 VL cases were identified (178 cases in the intervention area; 48.04/10,000/year) (377 cases in control areas; 107.95/10,000/year). The effect of the intervention was strongly significant. The estimated reduction of VL incidence rate by the intervention was 46.80% (p<0.0001). Another cohort study in Sudan found that the number of cases reported by village and month was significantly reduced following ITN (insecticide-treated 156-mesh) provision at all 4-month time points up to 20 months post-intervention. The greatest effect was detected 17–20 months post-intervention, with the number of cases on average reduced by 59%. The predicted number of cases in each village and each month in the absence of bednets from June 1999 to January 2001 was 3863, which compares of an observed number during that time of 2803. The investigators estimated that bednet intervention could have reduced the number of cases up to January 2001 by 1060, with a calculated protective effect of 27.4%. [10]

For the primary outcome, being the number of incident *L*. *donovani* infections as measured by seroconversion with the direct agglutination test at 12 and 24 months after the intervention, the risk of seroconversion during the two-year follow-up was significantly different between countries: 7.2% in India (529/7368) and 3.1% (163/5323) in Nepal [21]. The overall risk of seroconversion in the intervention (5.4%) and control (5.5%) groups was similar. At cluster level, the risk of infection was reduced by 10% in the intervention clusters compared with control clusters, but this effect was not significant. Longlasting insecticidal nets seemed to have an opposite effect on seroconversion in India and Nepal, but the interaction was not significant.

## Discussion

### Discussion of key results

Our systematic review has demonstrated that vector control interventions involving insecticide treated nets work effectively in community settings to reduce sandfly density, their exposure to and occurrence of VL among humans in South Asian (India, Bangladesh and Nepal) settings, as well as in other regional settings including in sub-Saharan Africa (Sudan), Latin America (Brazil) and the Mediterranean region (Turkey). Both insecticide impregnated bednets and commercially treated long lasting insecticide treated nets showed some levels of efficacy in the different interventions, with clear dose-response relationship that waned over time. While the community members well accepted the process of impregnating bed nets with slow releasing KO Tab 1-2-3, there could be problems with early erosion of insecticides resulting in effects lesser than expected. These problems did not happen with LLINs, but they could cost more requiring additional resources to ensure the sustainability of their widespread use. The review also found that combining ITNs and another vector control technique would work better than going with either of them separately.

For insecticide impregnated bednets, they had profound effects on sandfly density with its immediate reduction by as high as around 90% during the 2–4 weeks time period after impregnation. This high effectiveness observed in India did not replicate in Nepal. It was hypothesized that other vector control activities that were operating in the communities had reduced the vector density and burden, allowing little room for ITNs to show their effects [30]. In Bangladesh, the community had to wait as long as 5 months after the intervention to see its beneficial effect, which lasted through 18 months with 60% reduction in vector density. It is not clear what resulted in such a delay in receiving the benefits of insecticide impregnated bednets and weather the seasonal variations of the density of sandfly population and thus VL influenced by changes in the ambient weather condition played any role into that [35]. In another multi-country cRCT involving Bangladesh, India and Nepal, the effect of KO Tab 1-2-3 impregnated bednets did not last beyond 9 months. The investigators suggested that this could be due to operational errors during bed net impregnation and the breakdown of insecticide in nets over time [20]. This apparent weakness of impregnation of bed nets required further attention and support, especially in resource limited settings where this could be the most sustainable and cost-effective way of going with ITNs [15]. In other regional settings like Brazil, effects of deltamethrin impregnated bednets on *Lutzomyia longipalpis* showed reduction of both sandfly collection and landing under nets significantly. Mortality of *P*. *argentipes* was also found to be as high as 75% at 1 month after impregnation in Bangladesh. This effect lasted for a year with 50% sandfly mortality rate. It is important to consider that the effectiveness of ITNs and LLINs interventions were measured with sandflies densities, which has certain level of limitations since most of the included studies did not assess VL incidence as an outcome for the intervention’s effectiveness.

Insecticide impregnated bednets were also perceived to be well protective by the majority of the community members in Bangladesh soon after their introduction. But this perception changed over time along with the reduction of their effects and increase in sandfly nuisance. A small proportion of the community members also reported transient itching and unpleasant smell associated with bednet impregnation. Studies from South Asia as well as from other regional areas have shown significant reduction in human exposure to sandfly bites with this intervention. In Sudan, sandfly biting came down to zero with the use of bednets impregnated with insecticide lambda-cyhalothrin. The duration of exposure to sandfly bites varied by gender, with males being more at risk, and by age group, with under-five children being least exposed. Sleeping behaviour influenced by seasonal and temperature variation also played a significant role in the proper utilization of ITNs in the community. Further investigations are thus warranted to understand the socio-demographic, environmental and behavioural factors that influence the use of ITNs. Although this is the exposure of the insecticides that reduces the burden of sandflies and thus the occurrence of VL, interventions involving ITNs will not achieve its optimal utilization without taking the local contexts into account.

Regarding human VL cases, interventions with impregnation of bednets with KO Tab 1-2-3 in Bangladesh has shown around 70% reduction in VL-affected households. In another study from Bangladesh using retrospective cohort design, the estimated reduction of VL incidence rate by the intervention was 47% (p<0.0001). The occurrence of VL also came down by almost 60% with a 20 months long lasting effect when commercially treated LLINs were used. This outcome was measured in a cohort study in a different setting in Sudan. Unlike the other recent studies, a paired cRCT from India and Nepal with the intervention using LLINs did not find any significant difference in the risk of seroconversion over 24 months nor in the risk of clinical visceral leishmaniasis, even after adjusting for covariates. Although there were methodological differences between the studies testing similar interventions but in different population, that did not entirely explain the large variations in study findings.

The longer lasting effect of LLINs without needing to treat them in the community make them the ITNs of choice, given sufficient resources are there to roll out at the population level. In the intervention studies, LLINs were distributed free of cost, which would not be the case in a real-life setting involving the entire population. Otherwise, more than one-third of distributed PermaNet 2.0 was found in intact condition even after 22 months of use in India. Although, the average concentration of deltamethrin in the treated nets dropped substantially (from 24 to 9.5 mg/m^2^) over 18 months, as found in a study in Bangladesh, when comparing to IRS as a vector control measure, this reduction happened even faster with IRS when tested in India [15]. Regarding sandfly mortality, in a longer follow up study in Bangladesh, mortality on K-O TAB 1-2-3 impregnated nets dropped from 88.37% at 3 months to 69.12% at 20 months after use. In the same study, morality for LLIN was also tested and found to remain as high as 83% at 20 months of use.

The choice of an appropriate LLIN also depends on the mesh size and its border. Although showed significant and highest (91%) reduction in gravid *P*. *argentipes*, the Permanet 196 mesh/inch^2^+75 cm border was least preferred (as compared to 156 mesh/inch^2^ and 196 mesh/inch^2^) by only around half of the participants as it did not provide sufficient air circulation at night. The same should also apply to insecticide impregnated bednets, although if the community members treat bednets that they are currently using, they might be used to it and the mesh size might not become an issue for them then.

When compared with IRS, LLINs (PermaNet 3.0) was found to demonstrate stronger individual effect with monthly observation of percent reduction of sandfly density by 60.18–97.01%. The effect combining LLINs and IRS was the strongest (93.59–100%), as found in this Indian study [34]. In another study in Bangladesh, evaluating combined interventions, both LLIN and KOTAB showed similar effectiveness when combined with IRS (maximum around 85%) [28]. When LLIN was combined with outdoor spraying of vector breeding sites with relatively cheaper Chlorpyrifos 20EC [28], it was found to be the most effective in reducing *P*. *argentipes* sand fly density throughout the study period at a highly significant level (26.0% to 86.0%).

Given the current low incidence of VL in the endemic countries in SEA, as an outcome of the elimination campaign, it is now important to identify the suitable resource-efficient vector control methods to control for the disease. For ITNs, its widespread roll out at the community level could be replaced by targeted interventions in conjunction with IRS, along with case detection.

## Discussion of level of evidence and knowledge gap

Among the 25 studies included in this review, 23 were interventional studies. Of them, 10 were cRCTs showing robust evidence on the community effectiveness of ITNs including insecticide impregnated bed nets and LLINs on sandfly and VL control. Among the South Asian studies, seven were multi-country evaluations, which allowed for comparison of the effectiveness showing some real-life variations. There were also few follow-up studies generating longer term data from the same population. This allowed demonstration of the waning of insecticide impregnation in the bednets and their effects. From the studies that assessed the impact on ITNs on VL among humans, the majority were observational in nature and were conducted nested within other community interventions. These were efficient use of data and resources producing valuable population estimations. Surveys and qualitative interviews conducted alongside the interventions helped understand the community perception regarding the ITNs. Those studies provided contextual data needed for the successful implementation of the interventions.

There were some important limitations and knowledge gaps in the reviewed studies.

- In one hand, some studies were conducted under fairly controlled conditions which are not easily applicable in a national vector control programme. However, they provided indications of the way in which to undertake treatment programme. On the other hand, designing randomised control trials were also difficult given the unpredictability of the occurrence of VL in small areas, the lack of replicates limited the robustness of the findings. In the given context of very low case incidence as the positive outcome of the elimination campaign in South Asia, the organization of randomised trials was also deemed not feasible, which led to possible biased results. The effects of reduced vector densities on parasite transmission and new infections and cases could not be measured in recent studies as well. For one of the observational studies, incidence figures used in the epidemiological analysis were derived from clinics led by Doctors without Borders, and therefore only represented reported VL cases, and not true incidence. Moreover, the order with which villages were provided with ITN during the control campaign was significantly biased towards those with more reported cases.
- In several studies, bednet impregnation was carried out only in some target houses, and thus, the community effect was lost. There could also be reporting bias regarding washing/dipping of bednets with insecticide, as the information came from the household heads. The ITNs tested with bioassay were not randomly selected, resulting in possible underestimation of insecticidal effectiveness at the end of the trial.

The site-specific data regarding LLINs and environmental management had to be interpreted with caution because the limited number of clusters per arm provided uncertain estimates with wide confidence Intervals.

- For some studies, the intervention clusters had higher burden of sandflies. This could have underestimated the effect of ITN interventions. Other ongoing vector control activities, especially in the control arms, could also have diluted the effect of the ITN intervention provided through the study. The environmental factors (e.g., temperature, humidity) were not taken into account in most of the studies, although they could have played significant role on sandfly density over time, confounding the effectiveness of ITN interventions. Small sample size in some studies could result in findings with insufficient power. Disproportionate lost to follow-ups in the intervention and comparison clusters could have affected the study findings. The non-significance of the protective effect after 20 months post-distribution is probably a sample size issue, as there are fewer comparisons to make after 20 months. Moreover, clustering effect, especially when analysing number of female *P*. *argentipes* captured by aspiration in households post-intervention should have been taken into account.
- For the results regarding the protective efficacy of LLINs, it is important to consider that CDC light trap captures/monitor sand flies outside the bed nets, so that the actual protection for people sleeping under a LLIN from infective bites could be different. In addition, results based on aspiration captures in cattle sheds should be interpreted with caution as the number of *P*. *argentipes* collected was highly variable between and within clusters. The use of cattle sheds in the study may also have precluded an estimate of the absolute barrier effect of the different types of net since cattle sheds also constitute one of the probable breeding and resting sites for *P*. *argentipes* and some of the sand flies captured inside the nets may have newly emerged from the substrate beneath the net.
- It is important to consider that most of the included studies measured only vector density and mortality as entomological parameters. Therefore, several other entomological parameters are missing, such as entomological inoculation rate. In addition, several studies did not differ from male and female sandflies, which is important because only females are vectors.

## Conclusions

Our systematic review has found that both the insecticide impregnated bednets and the commercially treated long lasting insecticidal nets are effective in reducing the density of sandflies, although their effects on reducing burden of VL at the population level have not been researched extensively. Efficacy of both have strong dose response relationships and wane over time. While insecticide impregnated bednets are well accepted in the community, there could be problems that can lead to early erosion of insecticides from the nets. Long lasting insecticidal nets treated commercially do not have that issue but can demand additional resources to sustain their roll out in the community. Insecticide treated nets can even perform better along with indoor residual spraying. Combining comparatively low-cost insecticide impregnated bed nets with indoor residual spraying could be the way forward to controlling visceral leishmaniasis in resource limited settings. Given the current low incidence of VL in Southeast Asia, the effects of vector control measures are difficult to assess on the incidence of the disease. To ensure efficient resource allocation in the post-elimination era, efforts should be made to deliver the vector control interventions in conjunction with case detection in the community.

## Supporting information

S1 Text PRISMA Checklist. (DOCX)

S2 Text PRISMA Flow Diagram. (DOCX)
