# Supplementary material for: Efficacy and community-effectiveness of insecticide treated nets for the control of visceral leishmaniasis: A systematic review
Source: PLoS Negl Trop Dis. 2022 Mar 2;16(3):e0010196. doi: 10.1371/journal.pntd.0010196 (PMC8890655; doi:10.1371/journal.pntd.0010196)
Supplement: S2 Text — (DOCX) [file pntd.0010196.s002.docx]

S2 Text. PRISMA Flow Diagram

**Identification of studies via databases and registers**

Hits retrieved by database and using the database-specific search strategy and assessed by title/abstract (N=6331) (considering only the first 200 hits for google scholar)

Pubmed: 261

Science Direct: 234

Google scholar: 44652 (of which 12x200 have been assessed sorted by relevance)

LILACS: 08

WHOLIS: 00

WHO-IRIS: 3219

PAHO-IRIS: 209

**Identification**

Studies of potential relevance for vector control assessed by abstract (n=354):

Pubmed: 138

Science Direct:31

Google Scholar: 183

Lilacs:00

WHOLIS:00

WHO-IRIS: 02

PAHO-IRIS: 00

318 duplicates removed

**Screening**

One additional article recommended by an expert and that met the inclusion criteria was added

Reports assessed for eligibility

(n = 37)

After full application of inclusion and exclusion criteria, further 12 articles excluded:

-Not assessing ITNs as a vector control method (n=09)

-No primary research (n=03)

Studies included in review

(n = 25)

**Included**
